# Boosting LLM-assisted diagnosis: 10-minute LLM tutorial elevates radiology residents’ performance in brain MRI interpretation

**DOI:** 10.1007/s00234-025-03664-4

**Published:** 2025-06-04

**Authors:** Su Hwan Kim, Severin Schramm, Jonas Wihl, Philipp Raffler, Marlene Tahedl, Julian Canisius, Ina Luiken, Lukas Endrös, Stefan Reischl, Alexander Marka, Robert Walter, Mathias Schillmaier, Claus Zimmer, Benedikt Wiestler, Dennis Martin Hedderich

**Affiliations:** 1 Institute of Diagnostic and Interventional Neuroradiology, Technical University of Munich, Munich, Germany; 2 Institute of Diagnostic and Interventional Radiology, Technical University of Munich, Munich, Germany; 3 AI for Image-Guided Diagnosis and Therapy, Technical University of Munich, Munich, Germany

**Keywords:** Large language models, Human-AI interaction, Magnetic resonance imaging, Brain, Differential diagnosis

## Abstract

**Purpose:**

To evaluate the impact of a structured tutorial on the use of a large language model (LLM)-based search engine on radiology residents’ performance in brain MRI differential diagnosis.

**Methods:**

In this study, nine radiology residents determined the three most likely differential diagnoses for three sets of ten brain MRI cases with a challenging yet definite diagnosis. Each set was assessed (1) with the support of conventional internet search, (2) using an LLM-based search engine (© Perplexity AI) without prior tutorial, or (3) using the LLM-based search engine after a structured 10-minute tutorial. Reader responses were rated using a binary and numeric scoring system. Reading times and confidence levels (measured on a 5-point Likert scale) were recorded for each case. Search engine logs were examined to quantify user interaction metrics, and to identify hallucinations and misinterpretations in LLM responses.

**Results:**

Radiology residents achieved the highest accuracy when employing the LLM-based search engine following the tutorial, indicating the correct diagnosis among the top three differential diagnoses in 62.5% of cases (55/88). This was followed by the LLM-assisted workflow before the tutorial (44.8%; 39/87) and the conventional internet search workflow (32.2%; 28/87). The LLM tutorial led to significantly higher performance (binary scores: *p* = 0.042, numeric scores: *p* = 0.016) and confidence (*p* = 0.006) but resulted in no relevant differences in reading times. Hallucinations were found in 5.1% of LLM queries.

**Conclusion:**

Our findings demonstrate the considerable benefits that even low-effort educational interventions on LLMs can provide, highlighting their potential role in radiology training programs.

**Supplementary Information:**

The online version contains supplementary material available at 10.1007/s00234-025-03664-4.

## Introduction

Large language models (LLMs) are advanced artificial intelligence (AI) systems capable of processing and generating human language. Trained on vast amounts of text data, these models have demonstrated remarkable performance in various tasks across sectors [1].

With the rapid technological advancements of LLMs in recent years, numerous studies have explored applications of LLMs in radiological workflows. These include the definition of imaging protocols [2–4], performing differential diagnosis based on case presentations [5–7], error checking in radiology reports [8], generation of impressions in radiology reports [9, 10], information extraction from free-text radiology reports [11, 12] and more. Yet, despite the promising applications, the integration and adoption of LLMs in radiology is not without challenges. Data privacy concerns, bias and error propagation, lack of contextual understanding, and overreliance have been pointed out as relevant limitations of LLMs [13–15]. Against this background, the critical role of educating healthcare professionals on the appropriate use and potential pitfalls of LLMs has been emphasized [16, 17]. This may include training in prompt engineering, which is the process of crafting textual input strategically to optimise the LLM output [18].

One area where insufficient human oversight of LLMs could lead to clinical errors is radiological differential diagnosis. An earlier study on human-LLM collaboration in brain MRI differential diagnosis found that inadequate formulation of prompts can result in misleading LLM outputs, and lacking critical validation of LLM responses can lead to incorrect conclusions [19].

However, whether and how radiology readers can be trained to more effectively apply LLMs in radiological differential diagnosis has not been investigated yet. This study therefore aimed to evaluate the impact of a structured LLM tutorial on the performance of radiology residents in brain MRI differential diagnosis.

## Methods

Ethics approval and the need for informed patient consent were waived by the Ethics Committee of the Technical University of Munich.

### Study sample

**Fig. 1:**
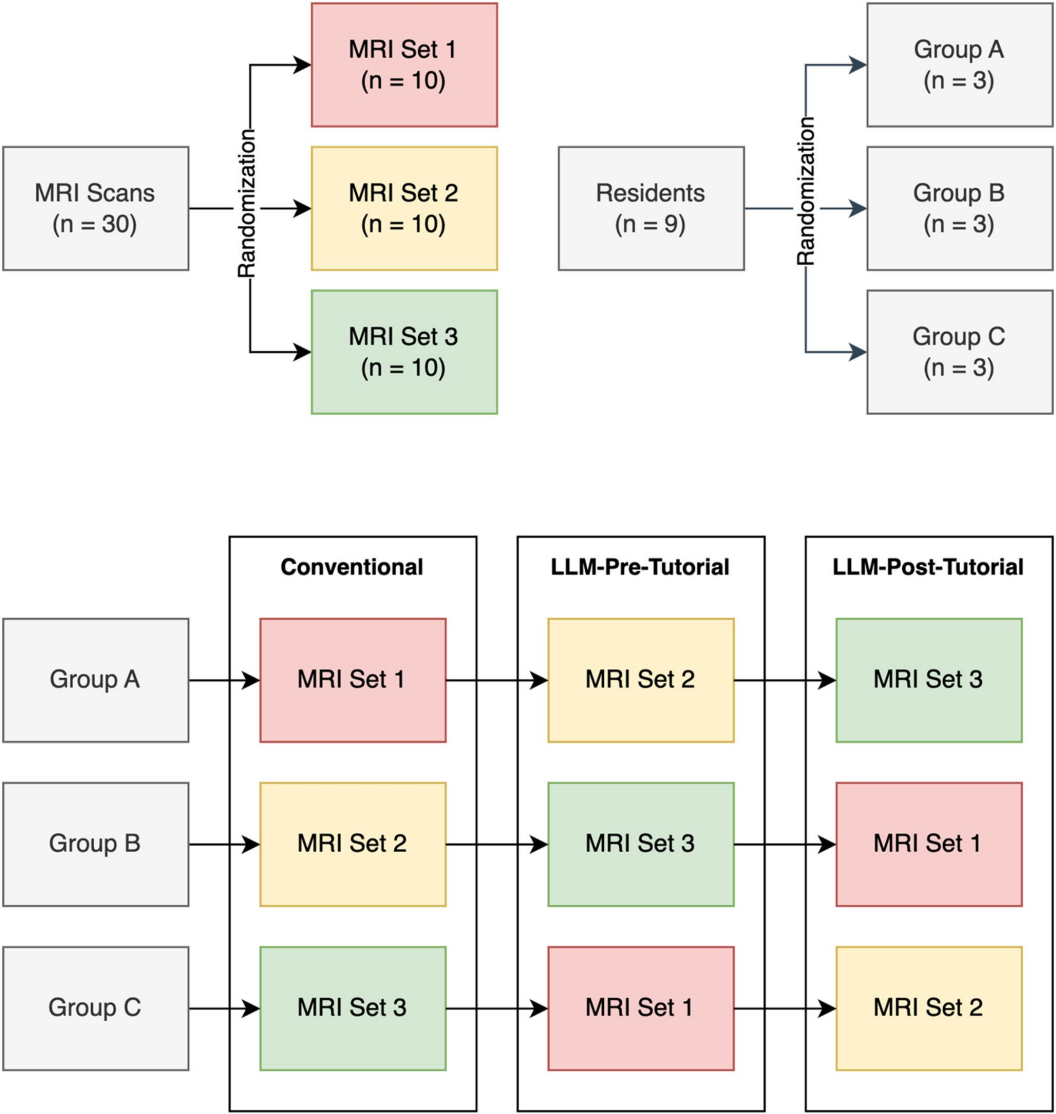
Study design

Thirty brain MRI exams acquired between 01/01/2016 and 12/31/2023 were selected from the local Picture Archiving and Communication System (PACS) system and randomized into three sets (Fig. 1). Included exams were deemed as sufficiently complex for use in radiological board certification exams by two board-certified neuroradiologists. Each exam featured an abnormal finding with a confirmed diagnosis, either histopathologically or through consensus between two neuroradiologists, taking into account all relevant clinical follow-up information. In each case, one or more arrows marked the image finding in question.

All thirty exams have been published previously [20]. The prior article evaluated the impact of varying multimodal input elements on the independent diagnostic accuracy of GPT4(V), whereas the present work investigates the diagnostic accuracy of radiology readers with LLM assistance. Twenty out of the thirty exams have further been included in another study [19]. While the previous study, like the present one, assessed the diagnostic performance of LLM-assisted readers, it involved a different group of readers, and did not provide any systematic training to the participants beforehand. A case overview is provided in Supplement 1.

In the present study, nine radiology residents with less than six months of dedicated experience in the neuroradiology division were recruited from the local radiology department and randomized into three groups (Table 1). The inclusion of only early residents ensured that most cases could not be solved by readers with prior knowledge alone, allowing for an investigation of the isolated effect of different web research workflows. Informed consent was provided by all participants.

**Table 1:** Overview of readers

Trait	Value
Total Number of Readers	9
Gender Distribution	7 Male (77.8%)/2 Female (22.2%)
Mean radiology experience (in months)	18.56 ± 15.93
Mean neuroradiology experience (in months)	2.22 ± 2.05
Readers who have used LLMs before	6/9 (66.7%)
Readers who have used LLMs for diagnosis before	1/9 (11.1%)

### Study design

Over the course of three sessions, each reader assessed three sets of ten brain MRI cases with varying workflows and provided up to three differential diagnoses for the annotated image findings, ranked by likelihood. Each case was reviewed only once per reader. To control for the confounding effects of case difficulty, each set of cases was assessed by the same number of readers with each workflow (Fig. 1). For every case, demographic information and a condensed medical history were provided. Sessions took place between 01/03/2024 and 22/05/2024.

First, conventional internet research was conducted to support differential diagnosis, either using web-based search engines, e.g. Google Search, or directly accessing trusted websites (Conventional). Second, readers utilized an LLM-based search engine (© Perplexity AI Inc., San Francisco, USA) but didn’t receive any training beforehand (LLM-Pre-Tutorial). PerplexityAI had been chosen as LLM interface for its ability to access real-time web content and provide source citations. Search queries were powered by GPT-4-Turbo (Generative Pre-trained Transformer 4 Turbo) by OpenAI. Third, another subset of ten cases was evaluated with the assistance of PerplexityAI. This time, however, the session was preceded by a structured tutorial on how to effectively use the tool (LLM-Post-Tutorial). Tutorial details are provided below. In both LLM-assisted workflows, participants were allowed to conduct additional internet search to validate LLM suggestions.

Reading times were recorded using a time tracking software (Toggl Track, © Toggl OÜ, Talinn, Estonia) by SHK and JW. Confidence levels were documented for each case on a 5-point Likert scale (1: not at all confident, 5: very confident). Following the second and third session, readers completed questionnaires to evaluate the experience with the LLM-assisted workflow.

### LLM tutorial

In a short tutorial of no more than 10 min, readers were given tips on how to effectively utilize the LLM-based search engine. The content of the tutorial was based on two earlier studies on the application of LLMs for brain MRI differential diagnosis. One study evaluated the contribution of varying multimodal input elements on the diagnostic performance of GPT4(V) and identified the textual description of radiological image findings as the key element [20]. The other demonstrated superior accuracy of LLM-assisted differential diagnosis over a workflow supported by a conventional search engine, but also determined several pitfalls in human-LLM interaction [19]. The full script of the tutorial is provided below:

*“A detailed description of image findings is by far the most important factor for accurate LLM responses. The description should include details about location*,* contrast enhancement*,* morphology*,* size and more. An accurate description of the finding location is particularly critical*,* an inaccurate specification of the location can result in misleading suggestions.*

*Providing relevant information about the medical history can improve the accuracy of LLM responses. However*,* clinical information unrelated to the image finding might result in misleading LLM outputs. Therefore only clinical information deemed to be relevant for the image finding in question should be provided.*

*Uploading screenshots of key image findings can help improve LLM responses*,* although their effect is only marginal.*

*Use of connotative terminology can lead to bias and should be avoided (e.g. the term ‘juxtacortical’ is strongly associated with multiple sclerosis).*

*Instructions can be made regarding the extent (number of differential diagnoses mentioned) and format (bullet points*,* table) of the LLM output.“*.

Readers were further encouraged to use the following prompt template:

*“You are a senior neuroradiologist. Below*,* you will find information regarding a brain MRI scan. Based on this information*,* identify the three most likely differential diagnoses*,* ranked by their likelihood. Present your findings in a table format with the following columns: ‘Rank’*,* ‘Differential Diagnosis’*,* and ‘Explanation’.*

*[Medical history]*

*[Image description]”*

Effective use of prompt templates was demonstrated to readers in two exemplary brain MRI cases (not included in the test sample).

### Sample size calculation

Based on previous research on the impact of LLM support on diagnostic performance of radiology readers [19], an effect size of 0.3 was assumed. Using a statistical power of 80%, an α error probability of 0.0167 (adjusted from 0.05 using Bonferroni correction for multiple comparisons), and a chi-square test, a minimum sample size of 182 was determined (G*Power, v3.1; [21]).

### Statistical analysis

To ensure performance did not solely depend on prior knowledge but the quality of research, cases where the correct diagnosis could be determined confidently without further research were excluded.

Accuracy of differential diagnoses was determined using two different scoring systems, as described previously [19] The first method used a binary scoring system, where responses were labeled as “correct” if the correct diagnosis was included among the submitted differentials, and “incorrect” if it was not. The second approach assigned scores ranging from 0 to 3 based on the rank of the correct diagnosis within the response (0: correct diagnosis not included, 1: correct diagnosis ranked third, 2: correct diagnosis ranked second, 3: correct diagnosis ranked first). Cases where a correct but less granular response was indicated were rated in consensus by two radiologists (1.5 and 2.5 years of dedicated experience in the local neuroradiology division). For binary scores, a chi-square test was initially applied across all groups, followed by pairwise chi-square tests. For numeric scores and confidence levels, a Kruskal-Wallis test was used to assess differences among all groups, with subsequent pairwise comparisons conducted using the Mann-Whitney U test. To control for false discovery rates, p-values were adjusted using the Benjamini-Hochberg procedure for both scores and confidence. Reading times were analyzed using an ANOVA test across all workflows, followed by pairwise t-tests. The significance level was set at *p* < 0.05. 5-point Likert-scale questionnaire results are reported using descriptive statistics.

Logs of the LLM-based search engine (Perplexity AI) were examined to quantify the number of queries and source references. Queries were categorized by query type (keyword searches vs. full-sentence instructions) and by content (differential diagnosis, radiographic features, sample images, anatomy, other). Sources were classified into journal articles and other online sources. The content of LLM responses were screened for incorrect or inconsistent information by two radiologists (1.5 and 2.5 years of experience in reading brain MRI exams) and confirmed by a certified neuroradiologist. As described previously [22], incorrect responses were classified into hallucinations (inconsistent with widely accepted radiological knowledge), misinterpretations (miscomprehending a question and giving contextually irrelevant replies), and clarifications (lacking comprehension of a prompt requiring its rephrasing). Radiographic features described in LLM responses were checked against reference articles of www.radiopaedia.org, which is a well-established source of radiological knowledge.

Data curation, analysis and visualization were performed using Python (version 3.9.7).

## Results

**Fig. 2:**
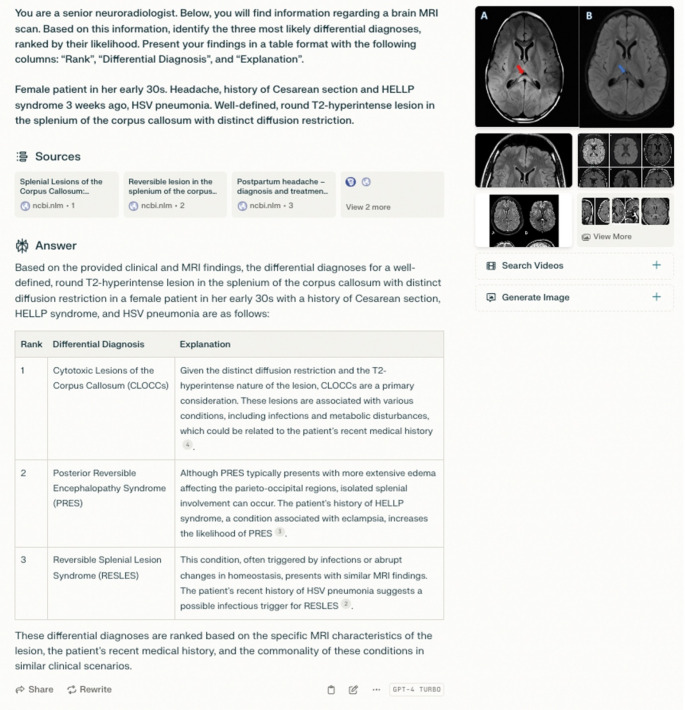
Screenshot of the PerplexityAI user interface. The correct diagnosis in this case was cytotoxic lesion of the corpus callosum (CLOCC)

The study involved a sample size of 270, which exceeded the calculated minimum sample size required for adequate power, as determined by our power analysis. 8 out of 270 responses (3.0%) were excluded from the analysis as readers were able to determine the correct diagnosis confidently without requiring further research. A sample LLM query and its results are shown in Fig. 2. 12 out of 262 cases (4.6%) required a consensus decision because the reader provided a correct but less specific diagnosis (e.g. “encephalitis” was counted as correct in a case of limbic encephalitis).

### Binary and numeric scores

**Fig. 3:**
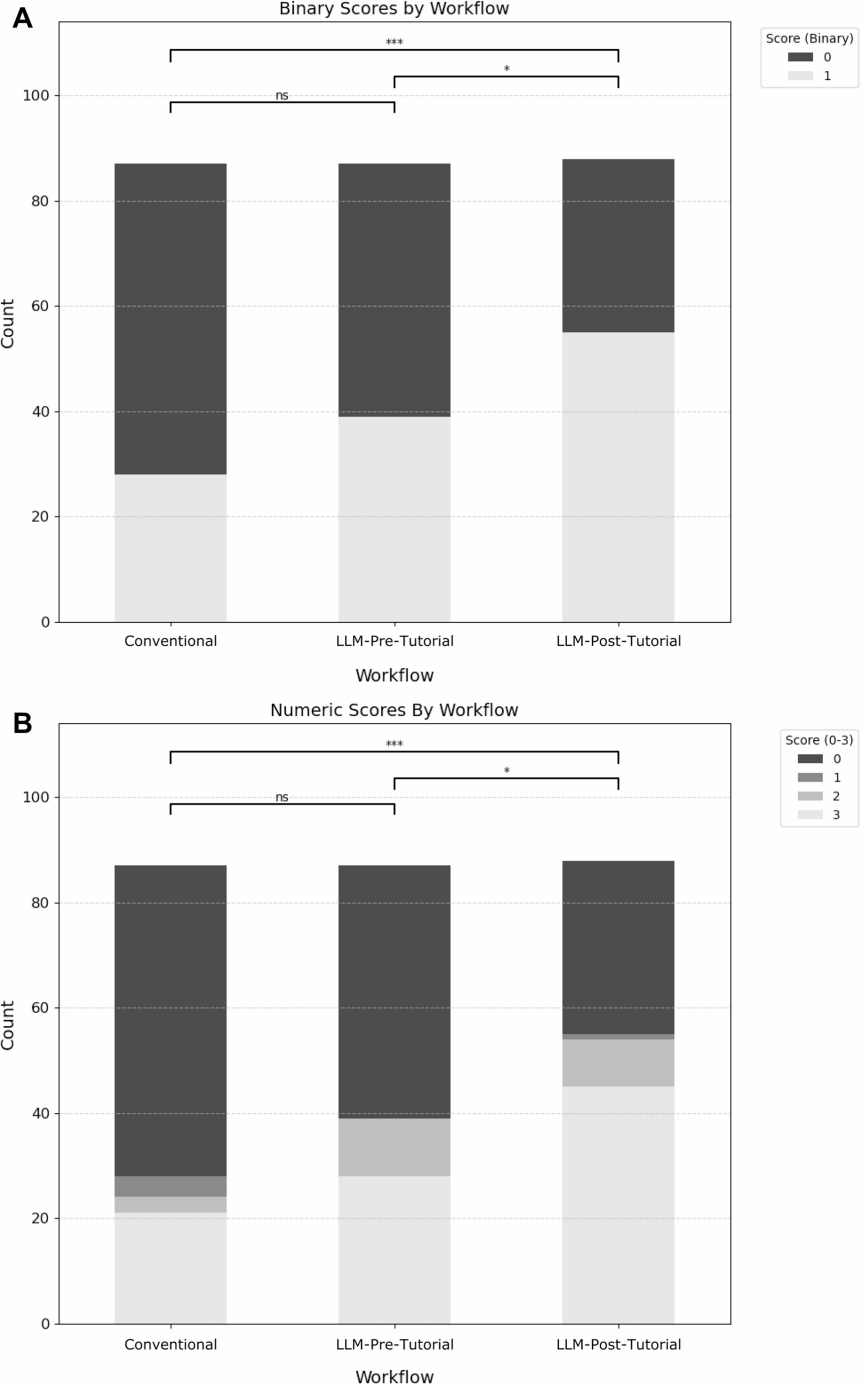
Diagnostic performance by workflow. **A**: Binary scores. Responses were classified as either correct (1) or incorrect (0). **B**: Numeric scores. Responses were assigned a score between 0 and 3, depending on the rank of the correct diagnosis within the response (3: correct diagnosis ranked first, 0: correct diagnosis not included in response). * *p* < 0.05. *** *p* < 0.001. ns: not significant

Based on the binary scoring system, 62.5% (55/88) of responses in the LLM-Post-Tutorial workflow were correct, compared to 44.8% (39/87) in the LLM-Pre-Tutorial and 32.2% (28/87) in the Conventional group (Fig. 3). An initial chi-square test across all groups indicated a significant overall difference (*p* < 0.001). Subsequent pairwise chi-square tests showed significant differences between LLM-Pre-Tutorial and LLM-Post-Tutorial (*p* = 0.042), as well as between LLM-Post-Tutorial and Conventional (*p* < 0.001), but not between LLM-Pre-Tutorial and Conventional (*p* = 0.119) (Table 2).

**Table 2:** Pairwise testing for inter-group differences in binary scores. Adjusted p-values have been corrected for a false-discovery rate of 0.05

Comparison	Chi2 Statistic	*p*	*p* (adjusted)
LLM-Pre-Tutorial vs. LLM-Post-Tutorial	4.81	0.028	**0.042**
LLM-Pre-Tutorial vs. Conventional	2.43	0.119	0.119
LLM-Post-Tutorial vs. Conventional	14.93	< 0.001	**< 0.001**

Comparison of numeric scores revealed a median score of 3 in the LLM-Post-Tutorial group, compared to 0 for both the LLM-Pre-Tutorial and Conventional groups (Fig. 3). The Kruskal-Wallis test confirmed significant overall differences among the workflows (*p* < 0.001). Pairwise comparisons using the Mann-Whitney U test revealed significant differences between LLM-Pre-Tutorial and LLM-Post-Tutorial (*p* = 0.016) and between LLM-Post-Tutorial and Conventional (*p* < 0.001), but not between LLM-Pre-Tutorial and Conventional (*p* = 0.092) (Table 3).

**Table 3:** Pairwise testing for inter-group differences in numeric scores. Adjusted p-values have been corrected for a false-discovery rate of 0.05

Comparison	U Statistic	*p*	*p* (adjusted)
LLM-Pre-Tutorial vs. LLM-Post-Tutorial	3049.5	0.011	**0.016**
LLM-Pre-Tutorial vs. Conventional	4267.5	0.092	0.092
LLM-Post-Tutorial vs. Conventional	5057.5	< 0.001	**< 0.001**

### Confidence

**Fig. 4:**
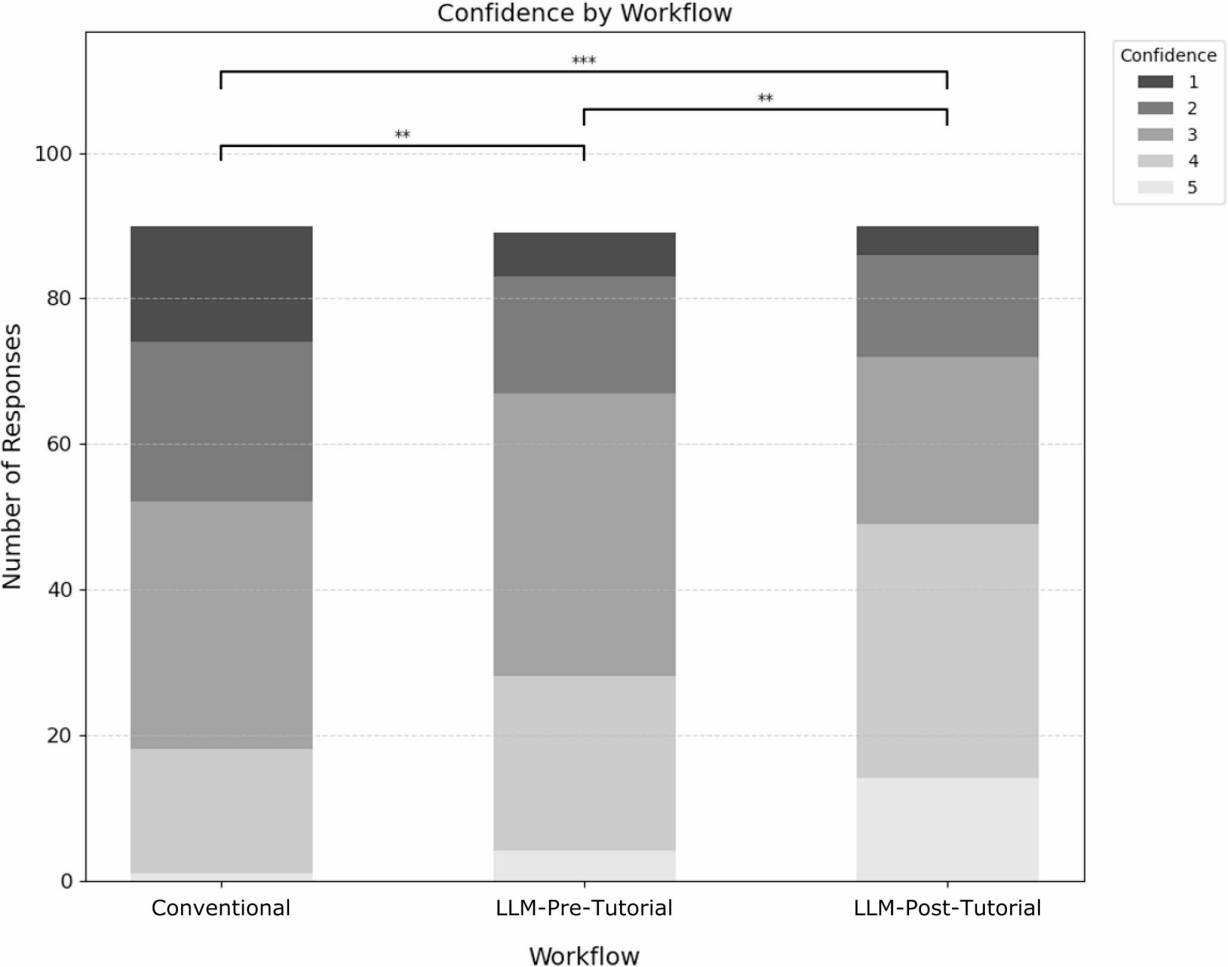
Confidence (5-point Likert scale rating) by workflow. ** *p* < 0.01. *** *p* < 0.001

Median confidence ratings were highest in LLM-Post-Tutorial (median = 4), followed by LLM-Pre-Tutorial (median = 3) and Conventional (median = 3). The proportion of high or very high confidence ratings (4 or 5) was 18% for Conventional, 31% for LLM-Pre-Tutorial, and 54% for LLM-Post-Tutorial (Fig. 4). The Kruskal-Wallis test showed a significant overall difference in confidence (H = 27.20, *p* < 0.001). Pairwise Mann-Whitney U tests revealed a highly significant difference between Conventional and LLM-Pre-Tutorial (*p* = 0.006), LLM-Pre-Tutorial and LLM-Post-Tutorial (*p* = 0.006) as well as between Conventional and LLM-Post-Tutorial (*p* < 0.001).

### Reading times

**Fig. 5:**
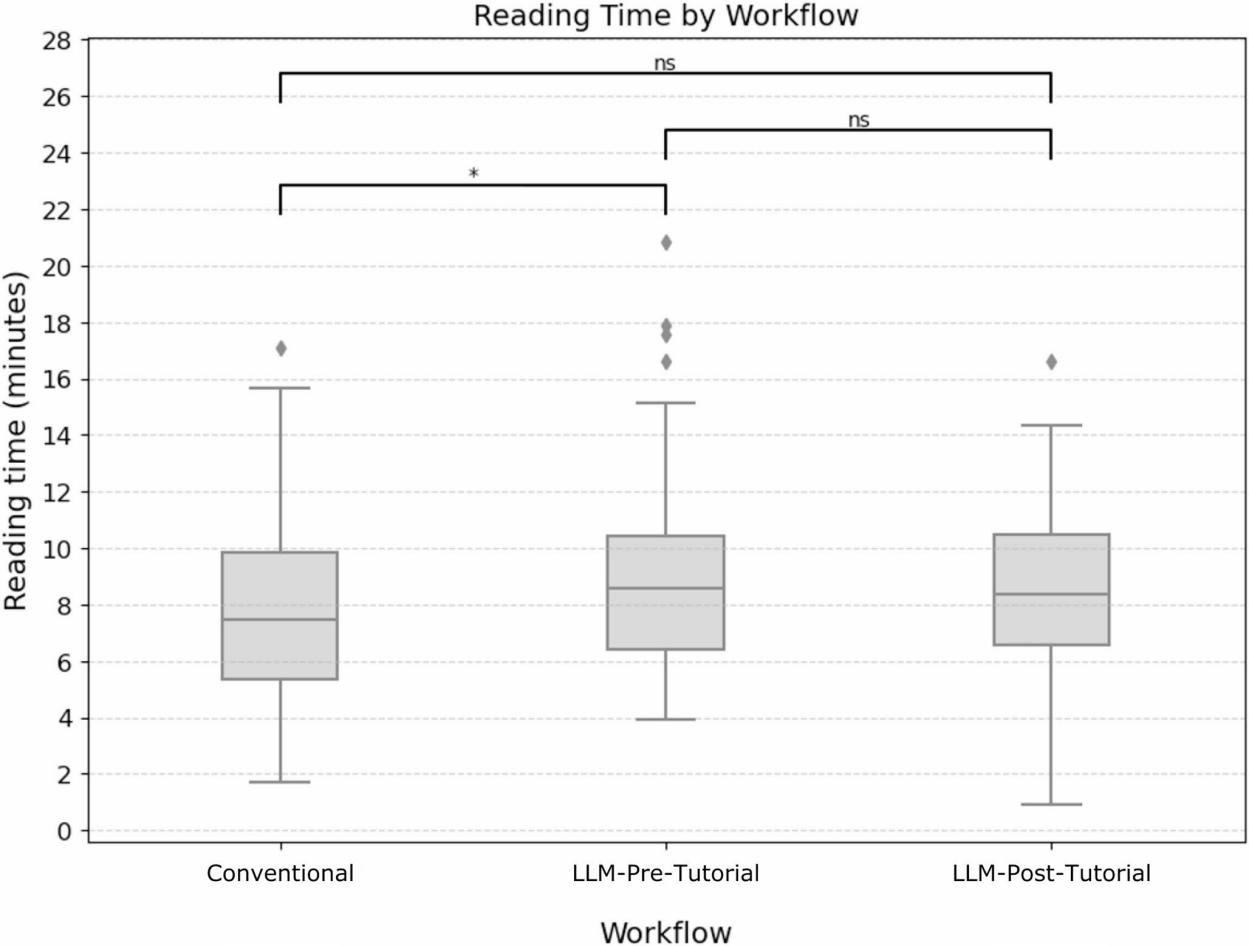
Reading time by workflow. * *p* < 0.05. ns: not significant

Mean reading times amounted to 07:43 min (Conventional), 08:59 min (LLM-Pre-Tutorial) and 08:35 min (LLM-Post-Tutorial) (Fig. 5). An ANOVA test showed a statistically significant overall difference (*p* = 0.030). Pairwise t-tests showed significant differences between Conventional and LLM-Pre-Tutorial (*p* = 0.013), but not between LLM-Pre-Tutorial and LLM-Post-Tutorial (*p* = 0.403) or between Conventional and LLM-Post-Tutorial (*p* = 0.069).

### Questionnaire results

The proportion of readers satisfied or very satisfied with the LLM-assisted diagnostic workflow increased from 55.6% (5/9) to 88.9% (8/9) following the tutorial. 55.6% (5/9) of readers indicated they would consider using the tool in clinical practice before the tutorial, compared to 88.9% (8/9) after the tutorial. 88.9% (8/9) of readers found the tutorial helpful or very helpful.

### Reader feedback and observations

Before the tutorial, most readers tended to use keyword-based queries rather than detailed instructions, similar to how conventional search engines operate. In general, the LLM tool was found particularly useful in generating an initial list of possible differential diagnoses to be evaluated through additional searches. In addition, the possibility to pose follow-up questions to initial query results was perceived as an advantage over conventional search engines. When using the provided prompt template, readers overwhelmingly appreciated the concise tabular format of the results which also included the rationale for the suggestion. However, some readers struggled to formulate accurate image descriptions, owing to their insufficient knowledge of neuroanatomy and brain MRI sequences.

### LLM response evaluation

A total of 413 LLM queries in 169 patient cases were examined (2.44 queries per case). In 11 out of 180 cases, LLM logs were not available because the user did not perform any LLM queries or because logs could not be retrieved due to technical errors of PerplexityAI. 5.8% of queries included incorrect inputs, such as inaccurate descriptions of finding locations or imaging characteristics. 7.3% of queries included screenshots of MRI findings.

The proportion of instruction-based queries increased substantially from 35.2 to 95.4% after the tutorial, while keyword queries decreased inversely from 64.8 to 4.6%. Whereas the majority of queries prior to the tutorial were classified as “Other” (58.4%), most queries following the tutorial were directed at relevant differential diagnoses (56.7%) and sample images of those (34.0%). Overall, 45.3% of sources indicated by PerplexityAI were peer-reviewed journal articles, with only minor differences between queries before and after the tutorial (49.2% and 40.6% each).

**Fig. 6:**
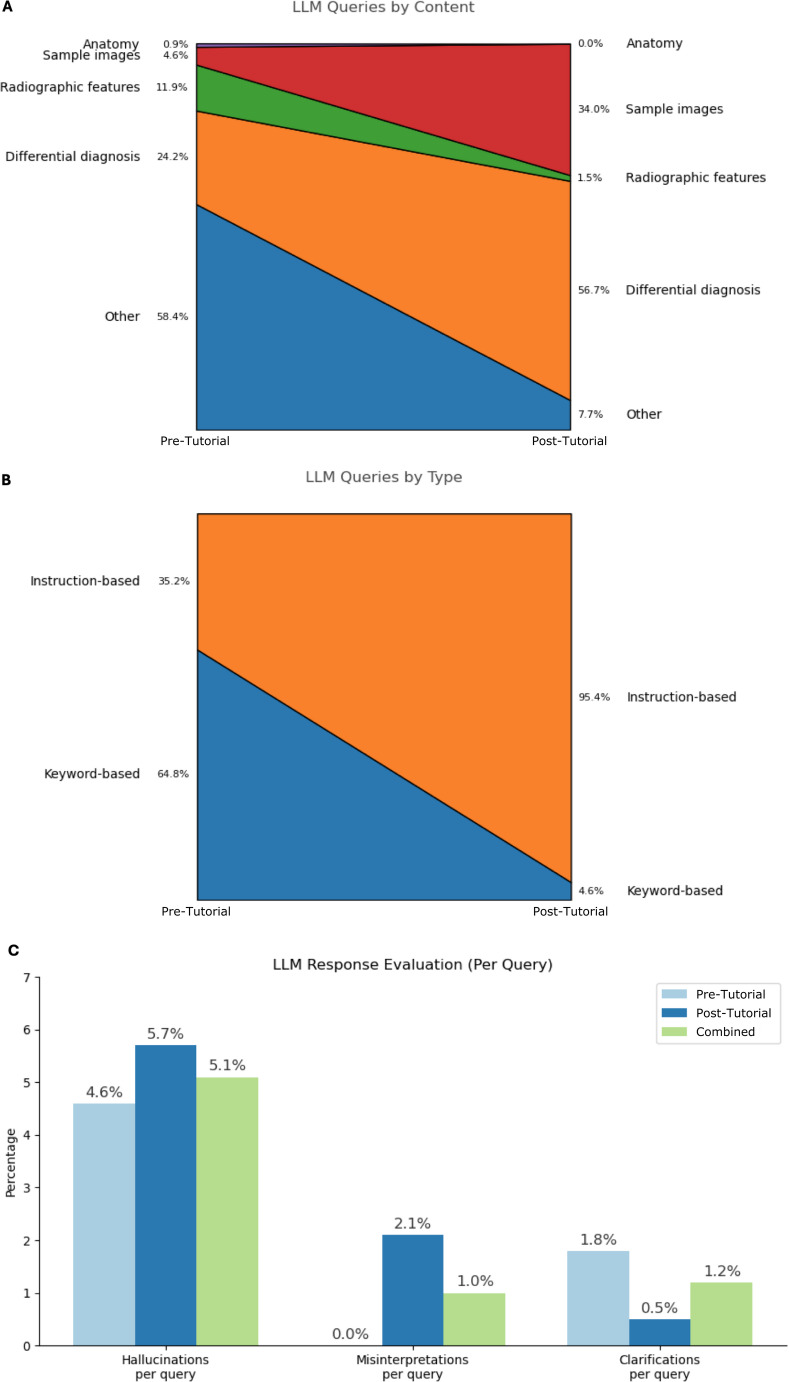
Evaluation of LLM queries and responses. A: LLM queries by content. B: LLM queries by type. C: Relative frequency of hallucinations, misinterpretations and clarifications per query

Hallucinations were observed in 5.1% of LLM queries (12.4% of cases; 20 responses in total). The LLM tutorial resulted in only a minor change in hallucination frequency (Pre-Tutorial: 4.6%, Post-Tutorial: 5.7%). 35.0% of hallucinations involved the misinterpretation of MRI screenshots provided as input or the LLM returning sample MRI images irrelevant to the context of the query. Hallucination details are provided in Supplement 2. Misinterpretations were found in 1.0% of queries (2.4% of cases), while clarifications occurred in 1.2% of queries (3.0% of cases) (Fig. 6).

## Discussion

This study evaluated the impact of a structured 10-minute LLM tutorial on the performance of radiology residents in LLM-assisted brain MRI differential diagnosis. We found that readers displayed higher performance, confidence levels and overall satisfaction after completing the tutorial. Compared to differential diagnosis supported by conventional internet search, both LLM-assisted workflows resulted in better performance, although only the Post-Tutorial workflow showed a statistically significant difference.

It is worth noting that the diagnostic accuracy in the LLM-Pre-Tutorial condition was not significantly higher than in the Conventional condition, unlike the findings of the precursor study [19]. One possible explanation is that participants in the earlier study were allowed to explore the LLM search engine and were provided with an example prompt prior to performing the LLM-assisted readings. Although they did not receive a comprehensive tutorial with detailed tips and instructions, this preliminary exposure may be considered a basic level of training - positioned between the Pre-Tutorial and Post-Tutorial conditions in the current study. Additionally, minor differences in diagnostic performance could be attributed to variations in case randomization and participant composition between the two studies.

Analysis of reader-LLM interactions revealed that following the tutorial, almost all queries were phrased as specific instructions, whereas most queries before the tutorial consisted of mere keywords, resembling conventional search engine queries. This observation is consistent with “Jakob’s Law” which is a well-known phenomenon in user experience (UX) stating that users prefer systems to behave like other familiar ones [23]. Similar to hallucination rates described previously [22], we found statements inconsistent with widely accepted medical knowledge in 5.1% of LLM responses. Many of these involved incorrect interpretations of MRI screenshots provided as input, confirming earlier studies demonstrating low performance of current state-of-the art LLMs in diagnostic tasks based on radiological images [24–27]. Interestingly, hallucinations were even found with PerplexityAI, which– unlike other Chatbots such as ChatGPT - combines LLMs with real-time information retrieval from the internet to support its answers with relevant sources [28]. Additional research is needed to develop further safeguarding measures against potentially harmful effects of hallucinations in clinical LLM applications. Notably, in 5.8% of queries misleading responses were generated not because of undesired LLM behavior, but due to incorrect finding descriptions provided by readers, emphasizing the essential role of conventional radiological skills in effectively employing LLMs for diagnostic tasks.

Our findings indicate that even minor, low-effort educational interventions on LLMs can yield remarkable outcomes, and support the notion that courses focused on the practical application of AI should become a core part of medical curricula and training programs [29–31]. Yet, given the novelty of the technology, validated educational content on the effective utilization of LLMs for specific clinical tasks is extremely scarce. The tutorial provided to the readers in this work was based on two previous studies specifically focusing on human-LLM collaboration and prompt engineering in brain MRI differential diagnosis [19, 20]. As the corpus of scientific evidence on LLM applications grows, medical societies should provide guidelines and courses on their appropriate use. Furthermore, platforms should be created to allow for healthcare professionals to exchange validated and effective prompts, similar to previous initiatives by radiological societies for sharing structured reporting templates [32, 33].

While LLM-assisted diagnosis shows promise, LLM integration into the diagnostic workflow must be approached carefully to avoid automation bias, to which novice radiologists are particularly susceptible [34, 35]. To preserve the development of independent clinical reasoning skills, readers should be encouraged to formulate their diagnostic impressions before consulting LLM outputs. Moreover, it is essential to caution users about the risk of hallucinations. Effective use of LLMs necessitates rigorous validation of model outputs - ironically reinforcing the need for foundational radiological expertise for their safe and effective adoption.

Unlike diagnostic performance, reading times did not improve with either of the LLM-assisted workflows. As prior work on AI-based image analysis algorithms and structured reporting has illustrated [36, 37], integration of technologies into the local IT infrastructure is critical for user acceptance and can boost efficiency. Vendors of radiology reporting solutions and Picture Archiving and Communication Systems (PACS) should explore ways to seamlessly embed LLM-based features supporting differential diagnosis and other tasks.

### Limitations

The following limitations need to be acknowledged.

First, only radiology residents with very little neuroradiology experience were included. This study design ensured that most cases could not be solved by readers with prior knowledge alone and allowed to investigate the isolated effect of distinct web research workflows, but limits generalizability of the findings to more experienced readers. Yet, our observation that inexperienced readers showed performance improvements ensuing the LLM tutorial despite struggling to formulate image descriptions suggests that moderately experienced readers, who are proficient enough to create accurate finding descriptions but not yet skilled enough to conduct differential diagnoses without assistance, might benefit even more.

Second, the findings in question were presented with annotations to isolate readers’ classification performance, but this approach reduced the realism of the scenario. It is possible that in actual practice, some of the more subtle findings would have been missed entirely.

Third, this study employed only a single LLM (GPT-4 by OpenAI) accessed through a specialized search engine (PerplexityAI). Future studies should compare several closed-source and open-source LLMs with respect to their utility in supporting radiology readers in differential diagnosis, including ones fine-tuned with domain-specific training data.

Fourth, this study did not evaluate downstream clinical endpoints. Given that resident-generated reports are routinely reviewed and approved by attending radiologists, the potential impact of integrating an LLM-based search engine on reporting efficiency, diagnostic turnaround time, and clinical decision-making warrants further investigation in future studies.

In conclusion, a concise but structured 10-minute LLM tutorial increased performance and confidence levels in LLM-assisted brain MRI differential diagnosis among radiology residents. These findings highlight the considerable benefits that even low-effort educational interventions on LLMs can provide.

## Electronic supplementary material

Below is the link to the electronic supplementary material.

Supplementary Material 1

## Data Availability

No datasets were generated or analysed during the current study.

## Declarations

The authors of this manuscript declare no relationships with any companies, whose products or services may be related to the subject matter of the article.

Informed patient consent was waived by the local Ethics Committee.

DMH is a member of the editorial board of *Neuroradiology*.
