# Supplementary material for: Boosting LLM-assisted diagnosis: 10-minute LLM tutorial elevates radiology residents’ performance in brain MRI interpretation
Source: Neuroradiology. 2025 Jun 4;67(8):2069–81. doi: 10.1007/s00234-025-03664-4 (PMC12494612; doi:10.1007/s00234-025-03664-4)
Supplement: Supplementary file 1 — Supplementary Material 1 [file 234_2025_3664_MOESM1_ESM.docx]

| **ID** | **Age Range** | **Sex** | **Correct Diagnosis** | **Condensed Medical History** |
| --- | --- | --- | --- | --- |
| 1.1 | 36 - 40 | F | Midline glioma | Intracranial mass in external CT scan, headache |
| 1.2 | 31 - 35 | F | ICA dissection | Recurring visual impairment on the left side, headache |
| 1.3 | 31 - 35 | M | Hemangioblastoma | Dizziness, headache |
| 1.4 | 41 - 45 | M | Chiari malformation type 1 | Right-sided headache, dizziness, right facial hypaesthaesia |
| 1.5 | 41 - 45 | M | Periorbital phlegmone | Periorbital swelling, headache, fever |
| 1.6 | 56 - 60 | F | Cerebral venous thrombosis | Acute desorientation |
| 1.7 | 51 - 55 | F | Chondrosarcoma | Newly diagnosed left sixth nerve palsy with diplopia, severe headaches for 2 days, left facial numbness for several weeks |
| 1.8 | 36 - 40 | F | Tumefactive multiple sclerosis (MS) | Sensory-motor hemiparesis left |
| 1.9 | 46 - 50 | M | Multinodular and vacuolating neuronal tumor (MVNT) | Subacute ptosis, right oculomotor palsy, diplopia |
| 1.10 | 36 - 40 | M | Cerebral cavernous venous malformation | Incidental finding in left temporal lobe in external imaging |
| 2.1 | 31 - 35 | F | Cytotoxic lesions of the corpus callosum (CLOCC) | Headache, history of Cesarean section and HELLP syndrome 3 weeks ago, HSV pneumonia |
| 2.2 | 61 - 65 | M | Cerebral amyloid angiopathy | Aphasia since yesterday, mild right hemiparesis |
| 2.3 | 41 - 45 | F | Echinococcosis | Headache, vomiting, mood disturbances Suspected brain abscess, patent foramen ovale |
| 2.4 | 41 - 45 | M | Pontine myelinolysis | History of alcoholic liver cirrhosis, suspicion of hyperactive delirium |
| 2.5 | 36 - 40 | M | Creutzfeldt Jakob disease | Gait ataxia, dysarthria, depression |
| 2.6 | 41 - 45 | M | Ecchordosis physaliphora | Incidental clival finding in external imaging, patient is asymptomatic |
| 2.7 | 51 - 55 | M | Miller-Fisher syndrome | Speech impairement since two weeks, double vision since three days, progressive swallowing disorder |
| 2.8 | 61 - 65 | F | Progressive multifocal encephalopathy (PML) | Progressive right-sided hemiparesis, history of chronic lymphocytic leukemia |
| 2.9 | 56 - 60 | M | Limbic encephalitis | Organic psychosyndrome for 3 months, psychomotor retardation, dysarthria |
| 2.10 | 41 - 45 | F | Spinocerebellar ataxia | Progressive gait ataxia, upper limb ataxia for 2 years |
| 3.1 | 61 - 65 | M | Superficial siderosis | Severe left sensorineural hearing loss |
| 3.2 | 21 - 25 | F | Moya Moya disease | Left hemiparesis for several weeks |
| 3.3 | 31 - 35 | M | Sarcoma | Scalp lesion DDx lipoma DDx atheroma, Pt. noticed swelling a few months ago, has grown since |
| 3.4 | 56 - 60 | F | Huntington's disease | Repeated orofacial dyskinesias, parathymic behavior. Generalized brain atrophy in CT. DDx neurodegenerative disease |
| 3.5 | 56 - 60 | M | Subependymoma | Incidental intracranial mass lesion in external images Patient is asymptomatic |
| 3.6 | 36 - 40 | M | Toxoplasmosis | Dysarthria, dysphagia, psychomotor slowing, suspected HIV infection, intracerebral lesions |
| 3.7 | 26 - 30 | M | Central neurocytoma | Headache and papilledema |
| 3.8 | 21 - 25 | F | Benign enhancing foramen magnum lesion | Ski injury with head impact, retrograde amnesia, initial confusion, subsequent improvement, MRI preferred due to young age |
| 3.9 | 56 - 60 | F | Posterior reversible encephalopathy syndrome (PRES) | Confusion, immunosuppression post-kidney transplant |
| 3.10 | 76 - 80 | M | Diffuse axonal injury | Recent history of high-speed trauma with traumatic SAH and hematopneumothorax |

Supplement 1: Case Overview.

| **Reader Prompt** | **Hallucinatory Output** | **Explanation** |
| --- | --- | --- |
| [Standard Prompt Introduction] Male in his 30s, scalp lesion differential lipoma differential atheroma, patient noticed swelling a few months ago and the lesion has grown since The brain MRI shows a large extradural lesion emerging from the dura of the left frontal lobe. It measures roughly 33 x 48 mm on the coronal view. The lesion expands out of the calotte, destroying the bone, and extends out of the skull. The skin is intact. The contrast image shows inhomogeneous gadolinium enhancement of the lesion. The lesion seems composed of cystic components. There is compressive effect upon the parenchyma of the left hemisphere. The midline is shifted slightly to the right. | "The inhomogeneous enhancement with gadolinium and the presence of cystic components are also characteristic features of meningiomas" | Meningiomas typically exhibit intense and homogeneous contrast enhancement. The LLM response indicates that inhomogeneous enhancement and cystic components are characteristic features of meningioma, which is incorrect. |
| [Standard Prompt Introduction] Female in her 20s, ski injury with head impact, retrograde amnesia, initial confusion, subsequent improvement The brain MRI shows a small roundly-shaped FLAIR hyperintense solid mass infratentorially anterior to the left cerebellar hemisphere. The contours are patchy. It is surrounded by cerebrospinal fluid. There is no diffusion restriction or microhemorrhage. The brain nerves are emerging from the brain stem in close proximity to the lesion but are not compressed. | "[…] The description of the lesion being surrounded by cerebrospinal fluid and its infratentorial location anterior to the cerebellum could suggest an arachnoid cyst." | Arachnoid cysts are not FLAIR-hyperintense (as indicated in the prompt). |
| [Standard Prompt Introduction] female in her 50s, confusion, immunosuppression following kidney transplant. Bilateral occipital FLAIR hyperintensities that do not involve the cortex. TOF MRA without any pathological finding. No diffusion restriction | "The symmetric FLAIR hyperintensities in the occipital lobes and cerebellum are typical of PRES, although the absence of diffusion restriction is less typical" | DWI signal is usually normal in PRES, restricted diffusion is presented in a quarter of cases (Radiopaedia). |
| Generate a list of differential diagnoses for a MRI scan of a patient with the following incidental imaging finding on brain MRI: [images of subependymoma in the left lateral ventricle] | "Based on the provided MRI images, which appear to show an incidental finding within the brain, a list of differential diagnoses could include: […] 3. Pineal cyst: A benign, fluid-filled sac located in the pineal gland in the brain. 4. Rathke's cleft cyst: A noncancerous cyst that occurs in the pituitary gland." | Based on two images showing an intraventricular lesion, the LLM mentioned "pineal cyst" and "Rathke's cleft cyst" as differential diagnoses, although the respective regions were not shown in the images. |
| Generate a list of differential diagnoses for a MRI scan of a patient with dysarthria, dysphagia and HIV and the following brain lesion: | "Progressive Multifocal Leukoencephalopathy (PML): Caused by the JC virus, PML can occur in people with severe immunodeficiency, including those with HIV/AIDS. MRI findings often show non-enhancing white matter lesions without mass effect." | The provided screenshot did not show any white matter lesions. |
| Generate a list of differential diagnoses for a MRI scan of a patient in his 30s with a headache and the following imaging finding: [image] | "Based on the provided MRI scan and the symptoms of a headache, here is a list of potential differential diagnoses: […] Cerebral venous sinus thrombosis: A blood clot in the brain's venous sinuses, which can cause increased intracranial pressure and headaches. Encephalitis: Inflammation of the brain, often caused by an infection. Multiple sclerosis: A disease of the central nervous system that can present with lesions in the brain and cause headaches." | In a case of an intraventricular lesion (central neurocytoma), several differential diagnoses not applicable to the intraventricular location are mentioned (cerebral venous sinus thrombosis, encephalitis, multiple sclerosis). |
| Generate a list of differential diagnoses for a MRI scan of a young female patient after skiing accident with the following imaging finding: [Image] | "[…] Ligament Injury: Injuries to the cruciate or collateral ligaments are common in skiing accidents due to twisting forces on the knee. Meniscal Tear: The menisci are cartilage structures in the knee that can be torn during accidents involving forceful twisting or rotation of the knee. Bone Contusion or Fracture: Direct impact or stress from an awkward fall can lead to bone bruises or fractures. Muscle Strain or Tear: The force exerted on muscles during a skiing accident can cause strains or tears, particularly in the quadriceps or hamstrings. Tendon Injury: Tendons, such as the patellar tendon, may be strained or torn if overstretched during an accident. [...]" | In a case of a foramen magnum lesion, multiple differential diagnoses not applicable to the location of the lesion are suggested (meniscal tear, bone contusion, muscle strain, patellar tendon injury, bursitis). |
| show an MRI example of Guillain-Barré Syndrome (GBS) where the facial nerve is enhanced |  | Images of spinal GBS manifestations are provided. |
| Bilateral diffusion disorder with FLAIR correlate in the dorsal corpus callosum | "Such MRI findings can occur in a variety of diseases and conditions, including Mild Encephalopathy with Reversible Splenial Lesion (MERS): a form of mild encephalopathy characterized by reversible lesions in the splenium of the corpus callosum. This disease is often associated with infections and can occur in children and adults. Cytotoxic Lesions of the Corpus Callosum (CLOCCs): This category includes various conditions that lead to cytotoxic edema in the corpus callosum, often associated with infections, metabolic disorders, or abrupt discontinuation of antiepileptic drugs. [...]" | Lists "Mild Encephalopathy with Reversible Splenial Lesion (MERS)" and "Cytotoxic Lesions of the Corpus Callosum (CLOCCs)" as two distinct differential diagnoses, although these are synonyms. |
| What are the reasons for diffusion disorders in the pons without contrast agent uptake? | "Mild Encephalopathy with Reversible Splenial Lesion (MERS): MERS is a rare disease characterized by reversible lesions in the corpus callosum, but can also affect other brain regions including the pons. These lesions show restricted diffusion without contrast uptake, indicating reversible intracellular swelling." | MERS / CLOCC is by definition located in the corpus callosum. The LLM response inaccurately indicates that it can be located in the pons. |
| which causes can lead to a bilateral diffusion disorder in the basal ganglia | "Movement disorders: The basal ganglia play a central role in the regulation of movement. Disorders such as restless legs syndrome, periodic movement disorders of the extremities and stereotypies can be associated with changes in the basal ganglia." | Movement disorders are possible clinical manifestations of basal ganglia pathologies, not the cause of basal ganglia pathologies. |
| Waller degeneration after chemotherapy in CLL | "Chemotherapy: The sources describe that chemotherapy-induced polyneuropathy (CIPN) is a common side effect of various chemotherapeutic agents. This form of neuropathy can result from the toxic effects of chemotherapeutic agents on nerve fibers. Although the sources do not explicitly mention Waller degeneration as a consequence of CIPN, it is plausible that the nerve damage caused by chemotherapy can trigger degenerative processes similar to those described in Waller degeneration." | The LLM response incorrectly describes that chemotherapy can trigger processes similar to those in Waller degeneration, while admitting not to have found a source for this information. |
| [Standard Prompt Introduction] Male patient in his 30s, no symptoms. The MRI shows multiple dot-like FLAIR hyperintensities grouped in the cortex of the left insula." | "Enlarged Virchow-Robin spaces can sometimes appear as dot-like hyperintensities on MRI. These are perivascular spaces that can be prominent in certain individuals and are generally considered a normal variant." | The lesions were described as FLAIR-hyperintense. The LLM response does not take into account that enlarged Virchow-Robin spaces would appear FLAIR-hypointense (isointense to CSF). |
| [Standard Prompt Introduction] male in his 40s, incidental clival finding in external imaging patient asymptomatic MRI features: T2 hyperintense, FLAIR iso to hypointense |  | When asked for sample images of clival differential diagnoses, the LLM presented images of a chondrosarcoma of the humerus. |
| you are a radiologist: MRI corpus callosum, FLAIR hyperintense, diffusion restricted lenticular lesion These are some pictures of the lesion: [images] | "The provided MRI images show various views of the brain, including axial, coronal, and sagittal planes." | No sagittal images were provided. |
| "you are a radiologist, female patient with vomiting, suspected brain abscess, headache. These are some MRI findings" [image] | "Based on the provided MRI image and the clinical history of a female patient with vomiting and headache, the MRI findings suggest the presence of a large encapsulated peripherally enhancing lesion within the brain. This lesion is characterized by an abnormal signal intensity on the right side of the brain, which could be located in the temporal or parietal lobe. The peripheral enhancement is indicative of a rim that is likely isointense to hyperintense on T1-weighted images and hypointense on T2-weighted images, as is typical for an abscess." | No post-contrast images were provided. The pathology is located on the left side, not right side. |
| "you are a radiologist; psychomotor retardation, dysarthria, organic psychosyndrome for 3 months, male patient in his 50s, mri findings are left side region of limen insulae, FLAIR hyperintense, T2 heme hyperintense, slight diffusion restriction, differential diagnosis" | "[…] The MRI findings described include left-sided FLAIR hyperintensity, T2 hyperintensity suggestive of hemorrhage (haem hyperintense), and slight diffusion restriction in the region of the limen insulae. These imaging characteristics help narrow down the potential causes." | "T2 heme hyperintense" does not indicate that there is hemorrhage. Hemorrhage typically shows low signal intensity in the T2 heme sequence. |
| [Standard Prompt Introduction] [severe left sensoineural heraing loss, male in his 60s] [cerebellum, intraVENTRICULAR FLAIR isointense tumor] | "The severe left sensorineural hearing loss and the presence of an intraventricular FLAIR isointense tumor in the cerebellum are highly suggestive of a vestibular schwannoma (acoustic neuroma), which is a benign tumor arising from the vestibular nerve sheath." | The LLM response suggests vestibular schwannoma as the most likely diagnosis for an intraventricular tumor in the cerebellum. However, vestibular schwannomas appear in the pontocerebellar angle, not within the ventricles. |
| [Standard Prompt Introduction]  male in his 30s, dysarthria, dysphagia, psychomotor slowing, intracerebral lesions, suspected HIV; FLAIR hyperintense, edema, multiple lesions, ADC hyperintense, peripherally enhancing, T2 hyperintense, white matter" | "Arachnoid cysts can occur in the posterior fossa near the fourth ventricle. They often appear as well-defined cystic lesions with rim enhancement on MRI, similar to the described findings. However, they usually do not demonstrate restricted diffusion." | Rim enhancement is highly unusual for arachnoid cysts. |
| [Standard Prompt Introduction] male in his 30s old dizziness headache cystic lesion in or close to the fourth ventricle contrast enhancement at the rims diffusion restriction" | "The FLAIR hyperintense lesion with contrast enhancement near the posterior horn of the left lateral ventricle, along with the sensory-motor hemiparesis on the left side, is highly suggestive of a lacunar stroke in the region of the posterior limb of the internal capsule. Lacunar strokes are small infarcts that typically occur in the deep brain structures, such as the thalamus, basal ganglia, or brainstem, and can cause pure motor, pure sensory, or mixed sensory-motor deficits." | Based on a lesion near the posterior horn of the left lateral ventricle, the presence of a lacunar stroke in the posterior limb of the internal capsule is suggested. These are two totally different anatomical structures. |

Supplement 2: Hallucinatory LLM responses. “Standard prompt introduction” indicates the following part of the prompt template provided to the readers: “You are a senior neuroradiologist. Below, you will find information regarding a brain MRI scan. Based on this information, identify the three most likely differential diagnoses, ranked by their likelihood. Present your findings in a table format with the following columns: ‘Rank’, ‘Differential Diagnosis’, and ‘Explanation’.”
